# Signs indicating dementia in Down, Williams and Fragile X syndromes

**DOI:** 10.1002/mgg3.430

**Published:** 2018-07-03

**Authors:** Oili Sauna‐Aho, Nina Bjelogrlic‐Laakso, Auli Siren, Maria Arvio

**Affiliations:** ^1^ KTO‐Special Welfare District of Varsinais‐Suomi Paimio Finland; ^2^ Departments of Child Neurology and Public Health University of Turku Turku Finland; ^3^ Special Services for Developmentally Disabled Tampere University Hospital Tampere Finland; ^4^ Department of Pediatric Neurology Kanta‐Häme Central Hospital Hämeenlinna Finland; ^5^ Department of Neurology Päijät‐Häme Joint Municipal Authority Lahti Finland; ^6^ PEDEGO University of Oulu Oulu Finland

**Keywords:** dementia, Down syndrome, Fragile X syndrome, intellectual disability, Williams syndrome

## Abstract

**Background:**

Intellectual disability (ID) and dementia reflect disturbed cortical function during and after developmental age, respectively. Due to the wide heterogeneity of ID population the decline in cognitive and adaptive skills may be different in distinct genetic subgroups.

**Methods:**

Using the British Present Psychiatric State–learning Disabilities assessment (PPS‐LD) questionnaire the dementia signs were screened in 62, 22 and 44 individuals (> 35 year of age) with Down (DS, OMIM number 190685), Williams (WS, OMIM number, 194050), and Fragile X syndrome (FXS, OMIM number 309550), respectively. The median age of those with FXS (59 years) was higher than of those with DS (50 years) and WS (53 years).

**Results:**

Most study participants with DS (80%) and FXS (89%) were or had been moderately or severely intellectually disabled while most participants with WS (73%) were or had been mildly or moderately disabled at adolescent age. The adolescent (premorbid) level of ID did not correlate with the dementia score. The median scores were 11/27, 1/27, and 0/27 in DS, WS, and FXS subgroups, respectively. Dementia that was confirmed by brain imaging, manifested as Alzheimer disease and as moya‐moya disease associated vascular dementia in DS and as vascular dementia in WS.

**Conclusions:**

This survey suggests that the risk of dementia varies depending on the cause of ID and that the severity of ID in adolescence does not predict the development of dementia at a later age. Consequently, the ID and dementia should be understood as separate clinical entities that need to be taken into account in the health management of intellectually disabled people. This is important for the arrangement of appropriate and timely interventions, which can be expected to delay the need for institutionalization.

## 1 INTRODUCTION

Intellectual disability (ID) and dementia reflect disturbed cortical function during developmental age and after developmental age, respectively. Alzheimer disease (AD) and vascular dementia are the most common dementia syndromes, both of which have a multifactorial etiology. Down syndrome represents the most common genetic, cerebral palsy‐ID the most common acquired, and autism spectrum‐ID the most common multi‐factorial ID syndrome. However, these three most common entities represent only 40%–55% of all people with IDs the remaining 45%–60% represent numerous smaller entities (Arvio & Sillanpää, 2003). In other words, intellectually disabled persons form an extremely heterogeneous population with different neuropsychiatric and somatic characteristics depending on the etiology of ID. Consequently, evaluation of dementia syndromes separately in different etiological ID subgroups can be considered clinically meaningful.

Intellectual disability itself bears many risk factors for cognitive impairment and dementia. Firstly, apart from disturbed cortical function, ID often associates with a structural cortical damage or dysgenesis. Secondly, nutritional factors form a double‐edged sword; overweight is common in certain genetic ID syndromes as is malnutrition resulting from swallowing difficulties or spasticity in some acquired ID syndromes. Thirdly, many persons with ID, especially those with autism spectrum disorders need polypharmacy because of their aggressive behaviors and other neuropsychiatric disorders and, thus, they may suffer from drug induced cognitive impairment. Finally, persons with ID may have a weak physical fitness simply because they cannot attend healthy cultural and sport activities without sufficient support and assistance from other people.

The national health care routines in Finland include etiological and neuropsychological evaluations that are done when a cognitive developmental delay is observed in children or when dementia is suspected in adults. Further, a licenced psychologist checks routinely the level of ID when an intellectually disabled person graduates, because such a statement from such an evaluation is needed for postgraduate studies and for disability allowance, which is financed by KELA (Social Insurance Institution of Finland). In accordance, the social security covers medicine costs only when the diagnosis of Alzheimer's disease has been confirmed by a brain magnetic resonance image or a computer tomography. Because of compliance problems, a brain imaging may not always be possible without sedation for patients with ID. Therefore, it is usually performed only in those cases when a cerebrovascular disorder or epilepsy is suspected in order to avoid risks related to anesthesia.

This study is a part of a larger project evaluating aging in the three most common genetic syndromes, that is, Down (DS, OMIM #190685), Williams (WS, OMIM #194050) and Fragile X (FXS, OMIM #309550), syndromes. DS (caused by trisomy 21) and WS (caused by hemizygosity at chromosomal site 7q11.23) affect equally both genders while FXS (caused by a mutation in the *FMR1* gene located on the long arm of the X chromosome) affects mainly males. Those with DS and WS represent 10%–15% and approximately 0.5% of intellectually disabled population, respectively, and males with FXS represent approximately 2% of all males with an ID (Arvio & Sillanpää, 2003). Many study subjects of this study have participated in our earlier prospective follow‐up and other studies (Arvio, 2016; Arvio & Bjelogrlic‐Laakso, 2017; Arvio & Luostarinen, 2016; Arvio, Peippo, & Simola, 1997; Eronen et al., 2002). For the present study, we recruited new individuals in order to compare the signs of dementia between these three genetic ID syndromes.

## 2 PATIENTS AND METHODS

### 2.1 Ethical compliance

The study was approved and reviewed by Pirkanmaa University Hospital District's ethics committee and a written informed consent was received from each participant or from their caretaker.

Finland is divided into 16 state‐supported regional full‐service districts for individuals who have ID. The authors (i.e., a neuropsychologist, 1st author and three neurologists), employees in different district organizations, assessed the study subjects and analyzed their medical records including data of brain scanning and the adolescent level of ID. For this study, all individuals (>35 years of age) with DS, WS, and FXS living in the South‐Häme district and all individuals with WS and FXS living in Pirkanmaa, Northern Ostrobothnia, and Southwest Finland districts were invited to participate. All 62 participants with DS, four of 22 with WS, and 19 of the 44 with FXS, have been included in our earlier studies (Arvio, 2016; Arvio & Bjelogrlic‐Laakso, 2017; Arvio & Luostarinen, 2016; Arvio et al., 1997; Eronen et al., 2002).

The British Present Psychiatric State‐Learning Disabilities assessment (PPS‐LD) was used for the screening of dementia signs. This PPS‐LD questionnaire includes 27 items and has been used in Finland as a tool in the dementia diagnostics, particularly among people who have ID (Arvio, 2016; Arvio, Ajasto, Koskinen, & Louhiala, 2013; Arvio & Bjelogrlic‐Laakso, 2017; Cooper, 1997a,b; Mölsä, 2001).

The relationship between the number of dementia signs (i.e., dementia score) and the chronological age was estimated by using regression models with the quadratic term of age. Bootstrap estimation was used to derive a 95% confidence interval of curvilinear correlation.

## 3 RESULTS

Clinical data of the three subgroups are presented in Table 1. The median age of those with FXS (59 years) was higher than of those with DS (50 years) and WS (53 years). Most study subjects with DS (80%) and FXS (89%) were or had been moderately or severely intellectually disabled while most individuals with WS (73%) were or had been mildly or moderately disabled at adolescent age (Table 1).

**Table 1:** Clinical data of the study subgroups

	Down	Williams	Fragile X
N (females/males)	62 (28/34)	22 (13/9)	44 (2/42)
Age range/mean/median, years	36–65/52/50	37–85/56/53	36–79/54/59
Severity of ID at adolescence			
Subnormal	0	1 (4.5%)	1 (2%)
Mild	11 (17.5%)	13 (59%)	3 (7%)
Moderate	26 (43%)	3 (14%)	17 (39%)
Severe	24 (37%)	4 (18%)	22 (50%)
Profound	1 (2.5%)	1 (4.5%)	1 (2%)

*Note*

ID: intellectual disability.

**Figure 1:**
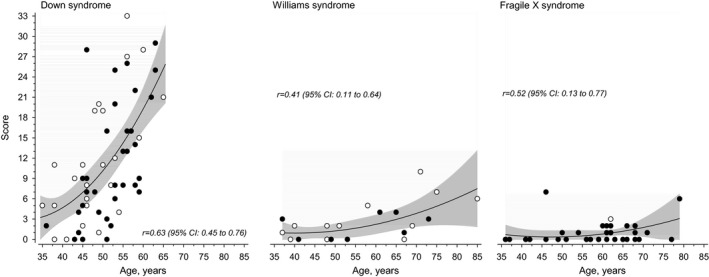
Dementia scores of three etiological subgroups according chronological age

Figure 1 shows dementia scores according to chronological age and Table 2 the division of 27 dementia signs in the three etiological subgroups. Those with DS had the highest scores (median score 11); reduced self‐care skills, loss of energy, impaired understanding, and forgetfulness were their most common signs (Table 2). In WS subjects (median score 1), the most common signs included weight change, change in appetite, onset or increase of physical aggression and reduced quantity of speech. Those with FXS, had the lowest dementia scores (median score 0).

**Table 2:** Signs indicating dementia in study subgroups

Sign	Down	Williams	Fragile X
Autonomic anxiety	16 (25%)	1 (5%)	1 (2%)
Change in appetite	8 (12.5%)	3 (14%)	1 (2%)
Changed sleep pattern	21 (35%)	2 (9%)	1 (2%)
Coarsening of personality	16 (25%)	1 (5%)	1 (2%)
Confusion	8 (12.5%)	0	0
Delusions	6 (10%)	1 (5%)	0
Diurnal mood variation	14 (22.5%)	1 (5%)	1 (2%)
Forgetful	28 (45%)	0	1 (2%)
Forgetting people′s names	5 (7.5%)	2 (9%)	0
Geographical disorientation	9 (15%)	0	0
Impaired understanding	34 (55%)	1 (5%)	1 (2%)
Irritability	19 (30%	2 (9%)	1 (2%)
Loss of concentration	21 (35%)	0	0
Loss of energy	37 (60%)	2 (9%)	1 (2%)
Loss of literacy skills	6 (10%)	1 (5%)	1 (2%)
Misery	16 (25%)	0	0
Onset of or increase			
In other maladaptive behavior	11 (17.5%)	1 (5%)	0
In physical aggression	11 (17.5%)	3 (14%)	1 (2%)
In verbal aggression	6 (10%)	1 (5%)	0
Of fearfulness	16 (25%)	1 (5%)	1 (2%)
Reduced quantity of speech	26 (42.5%)	3 (14%)	0
Reduced self‐care skills	40 (63.5%)	2 (9%)	1
Social withdrawal/			
Reduced social interaction	12 (20%)	0	0
Tearfulness	12 (20%)	0	0
Temporal disorientation	16 (25%)	3 (14%)	0
Weight change	25 (40%)	8 (36%)	0
Worry	16 (25%)	2 (9%)	1 (2%)

Table 3 presents the state of health of the subjects. All subjects with WS had co‐morbidities of which the most commonly occurring were hypertension, psychiatric disorder, hypercholesterolemia and congenital heart defect. Dementia was diagnosed in each case by typical symptoms and by brain imaging. In DS, it manifested as Alzheimer disease in all but one subject who had vascular dementia (presumably resulting from moyamoya disease and cerebral hemorrhage) and as vascular dementia in subjects with WS (presumably evolving from transient ischemic attacks). In addition, the neurologist in charge had prescribed acetylcholinesterase inhibitor or memantine to 14 other subjects with DS with without brain scanning, after excluding other disorders that may mimic dementia.

**Table 3:** State of health of the study subjects

	Down	Williams	Fragile X
No health problem	8 (13%)	0	3 (7%)
Neurological comorbidity			
Alzheimer disease^a^	14 (23%)	0	0
Epilepsy	15 (24%)	3 (14%)	12 (27%)
Vascular dementia, moyamoya^a^	1 (2%)	3 (14%)	0
Transient ischemic attacks	0	5 (23%)	1 (2%)
Sleep apnea	1 (2%)	2 (9%)	0
Migraine	0	3 (14%)	0
Movement disorder	1 (2%)	6 (27%)	0
Gastrointestinal comorbidity			
Diverticulitis	0	8 (36%)	0
Coelicia	1 (2%)	2 (9%)	0
Reflux	1 (2%)	6 (27%)	0
Constipation	5 (8%)	5 (23%)	0
Rectal prolapse	0	7 (32%)	1 (2%)
Hirschprung disease, stoma	1 (2%)	0	0
Cardiovascular comorbidity			
Congenital heart defect	7 (11%)	10 (45%)	1 (2%)
Hypertension	0	17 (77%)	2 (4.5%)
Autoimmune disease			
Diabetes type 1 or 2	3 (5%)	7 (32%)	3 (7%)
Hypothyroidism	31 (50%)	6 (27%)	4 (9%)
Pemphigoid	0	0	1 (2%)
Rheumatoid arthritis	0	2 (9%)	0
Psychiatric disorder	1 (2%)	11 (50%)	5 (11%)
Sensory impairment			
Visual impairment	1 (2.5%)	8 (13%)	2 (4.5%)
Cataracta	7 (11%)		3 (7%)
Chronic infection			
Skin infection	0	0	2 (4.5%)
Urinary infection	4 (6%)	2 (9%)	0
Skeletal impairment			
Scoliosis/kyphosis	1 (2.5%)	11 (38)	5 (11%)
Joint luxations	3 (5%)	2 (7)	0
Craniosynostosis‐cleft palate	0	1 (4.5%)	0
Other			
Hypercholesterolemia	4 (10%)	9 (41%)	2 (4.5%)
Gout	2 (5%)	0	0
Renal insufficiency	2 (5%)	1 (4.5%)	0
Enlarged prostate	1 (2%)	0	12 (29%)
Psoriasis	2 (5%)	0	0
Cancer	0	2 (9%)	0
Asthma	0	1 (3)	1
Lymphoedema in legs	0	3 (14%)	0

*Note*

a Confirmed with brain magnetic resonance image, or with brain tomography.

There was no connection between the adolescent (premorbid) severity of ID and dementia score (Table 4). The most common dementia signs among the three subjects (ages 50, 53, and 61 year) with a profound ID (as estimated at adolescence) were weight change, loss of energy and sleep disorder. The most common dementia signs of the four youngest DS subjects (ages 39, 42, 42, and 43 year) with mild ID (as estimated at adolescence) were irritability, worry, weight change, and reduced self‐help skills.

**Table 4:** Dementia scores according the adolescent severity of intellectual disability (ID), DS, WS, FXS

Severity of ID score	N	Ages or median age	Scores or median
Subnormal	2	29 (FXS) and 58 (WS)	0 and 5
Levis	27	54	2
Moderate	46	54	3
Severe	50	54	2
Profound	3	50 (DS), 53 (DS) and 61(FXS)	5, 8 and 1

*Note*

DS: Down syndrome; FXS: Fragile X syndrome; WS: Williams syndrome.

## 4 DISCUSSION

The centralized service system for intellectually disabled persons, since the 1960's, has made the scientific research of ID medicine possible in Finland. During the past decades the life expectancy of intellectually disabled persons has increased (Arvio, Salokivi, & Bjelogrlic‐Laakso, 2016; Arvio, Salokivi, Tiitinen, & Haataja, 2016; Westerinen et al., 2016). According to our national registries, 8% of persons with ID are nowadays senior citizens (aged 65+) while the respective percentage 20 years ago was 2% (Arvio, Salokivi, & Bjelogrlic‐Laakso, 2016). Still, there are not many DS persons aged over 65 as seen also in this survey, whereas one‐third of our WS subjects and one‐fourth of our FXS subjects were senior citizens (Figure 1). The same British questionnaire (as used in Finland since early 2000, 7–10) has been used as a measurement tool in all these assessments, which allows a reliable comparison of the occurrence of dementia signs in different ID syndromes.

Intellectually disabled persons form both etiologically and clinically an extremely heterogeneous group of people and the results of this study indicate that the etiology of ID has a significant prognostic role in the development of neurodegenerative dementia. The association between DS and Alzheimer disease has been known for a long time (Evenhuis, 1990, 1996; Golden, Nielsen, Pober, & Hyman, 1995; Head, Powell, Gold, & Schmitt, 2012; McCarron et al., 2017; Tyrrell et al., 2001) and as expected, the persons with DS were found to have the highest dementia scores. There is an earlier case report of a young WS patient showing neuropathological findings resembling Alzheimer disease (Shultz et al., 2004); but in this study three elderly WS subjects, not unexpectedly (with a history of transient ischemic attacks) suffered from vascular dementia. Almost no dementia signs were observed in FXS subjects although their median age was 9 years higher than that of the DS subjects. Thus, it seems reasonable to assume that the dementia development should be followed more carefully in DS and WS than in FXS so that the early signs of dementia are recognized in time in order to initiate appropriate interventions and, thus, to prevent a premature institutionalization.

On the other hand, the severity of ID (determined in adolescence) did not seem to predict the development of dementia at later age. In this study, the signs of dementia were found to be somewhat different in mild and profound ID. In the three subjects with profound ID, the most common dementia signs were weight change, loss of energy, sleep disorder, whereas among the four youngest patients with mild ID, the leading symptoms were irritability, worry, weight change, and reduced self‐help skills.

The results from earlier studies on dementia in intellectually disabled persons published by others cannot be compared directly with our results because of different study designs and different patient characteristics. For example, in the papers of Evenhuis and Shutz et al. (Evenhuis, 1990, 1996; Golden et al., 1995) the adolescence level of ID has not been defined and it has not been detailed how the study participants had been divided into demented and nondemented at the base line. Another confounding factor is related to grouping study participants to DS and non‐DS patients without defining the etiology of the latter group, which may represent numerous genetic, acquired and multi‐factorial entities and even progressive disorders. Taken the heterogeneity of ID population, no reliable conclusions on the risk and the onset age of dementia can be drawn without the known etiology of ID and without the determined best level of ID, which is usually reached at young adulthood.

In conclusion, the risk of dementia appears different in DS, WS, and FXS syndromes suggesting that ID and dementia should be managed as distinct clinical entities. Unlike general population, people with an ID have undergone several psychological assessments including intelligence testing during childhood and adolescence. This information combined with regular screening for dementia signs done by close‐care takers followed by neurological evaluations are the key to appropriate diagnostics and successful interventions to improve the quality of life of those with dementia signs. Further studies are needed to evaluate the occurrence of dementia in other disorders such as, e.g., autism spectrum disorder and cerebral palsy associated IDs, in order to see whether this same phenomenon does concern also the other than these three genetic ID syndromes.

## CONFLICT OF INTEREST

The authors declare no conflict of interest.
